# The Efficacy and Safety of Rivaroxaban for Venous Thromboembolism Prophylaxis after Total Hip and Total Knee Arthroplasty

**DOI:** 10.1155/2013/762310

**Published:** 2013-02-21

**Authors:** Robert D. Russell, William R. Hotchkiss, Justin R. Knight, Michael H. Huo

**Affiliations:** Department of Orthopaedic Surgery, University of Texas Southwestern Medical Center, 1801 Inwood Road, Dallas, TX 75390-8883, USA

## Abstract

Venous thromboembolism (VTE) is a common complication after total hip and total knee arthroplasty. Currently used methods of VTE prophylaxis after these procedures have important limitations, including parenteral administration, and unpredictable plasma levels requiring frequent monitoring and dose adjustment leading to decreased patient compliance with recommended guidelines. New oral anticoagulants have been demonstrated in clinical trials to be equally efficacious to enoxaparin and allow for fixed dosing without the need for monitoring. Rivaroxaban is one of the new oral anticoagulants and is a direct factor Xa inhibitor that has demonstrated superior efficacy to that of enoxaparin. However, the data also suggest that rivaroxaban has an increased risk of bleeding compared to enoxaparin. This paper reviews the available data on the efficacy and safety of rivaroxaban for VTE prophylaxis after total hip and total knee arthroplasty.

## 1. Introduction

Venous thromboembolism (VTE) is a common complication after total hip arthroplasty (THA) and total knee arthroplasty (TKA). Without anticoagulant prophylaxis, symptomatic deep venous thrombosis (DVT) occurs in approximately 15%–30% of the patients undergoing THA and TKA [1, 2]. Patients undergoing TKA are at higher risk for developing DVT; however, the rate of symptomatic DVT is higher after THA [1, 3, 4]. With evolving surgical technique, and methods of preventing VTE, the rate of VTE has decreased over time [1]. Using currently accepted methods of VTE prophylaxis, the rate of symptomatic DVT is approximately 1%–3%, and the rate of pulmonary embolism (PE) is approximately 0.2%–1.1% [2, 5–8]. The efficacy of VTE prophylaxis must be weighed against the risk of bleeding complications for the patients. The most commonly used VTE chemoprophylaxes after THA and TKA are low-molecular-weight heparin (LMWH), adjusted-dose warfarin with a targeted INR of 2-3, fondaparinux, or aspirin [2]. 

Current VTE prophylaxis regimens have significant shortcomings. Warfarin has a slow onset of action and has a narrow therapeutic window requiring frequent monitoring. Patients taking warfarin have only a 33% compliance rate and are frequently outside the targeted INR range increasing the risk of both bleeding and VTE [9, 10]. Low-molecular-weight heparin (LMWH) and fondaparinux must be administered parenterally, which requires time and cost. Patients are less compliant with administration of these drugs due to these barriers. One study reported only 75% continued the medication after discharge [9]. However, both warfarin and LMWH have acceptable efficacy and safety profiles. 

In addition to the type of VTE prophylaxis used, another important consideration is the duration of prophylaxis. The average time to diagnosis of symptomatic VTE after THA and TKA is approximately 21 days and 10 days, respectively [11]. The average duration of inpatient stay for patients undergoing THA and TKA is 3 and 4 days, respectively [9]. The current recommendations for the duration of prophylaxis are a minimum of 10 days, with extension up to 35 days for patients undergoing THA [2]. Thus, in addition to optimal efficacy and safety profiles, the ideal VTE prophylaxis regimen must be administered by the patient and requires no frequent monitoring. 

Three new oral anticoagulants have been developed. Dabigatran is a direct factor IIa (thrombin) inhibitor. Rivaroxaban and apixaban both are direct factor Xa inhibitors. Only rivaroxaban is currently approved in the United States for VTE prophylaxis after THA and TKA [2]. The results for apixaban and dabigatran have demonstrated similar efficacy and safety profiles when compared to enoxaparin, and these drugs have been approved in Canada and countries in Europe [12–17]. Randomized-controlled trials comparing rivaroxaban to enoxaparin for the prevention of VTE after THA and TKA have demonstrated superior efficacy of rivaroxaban to enoxaparin [18–21]. However, there is also evidence suggesting that rivaroxaban has an increased risk of bleeding complications compared to enoxaparin [22, 23]. While rivaroxaban appears to be a promising alternative to LMWH or warfarin for VTE prophylaxis after THA and TKA, the long-term safety of rivaroxaban remains to be determined. 

The purpose of this paper is to critically analyze the current data on the efficacy and safety of rivaroxaban after total hip and total knee arthroplasty.

## 2. Pharmacokinetics of Rivaroxaban

The recommended dose of rivaroxaban is 10 mg daily for 10 to 35 days for VTE prophylaxis starting 6–10 hours after THA or TKA [24]. Rivaroxaban is a competitive, direct factor Xa (FXa) inhibitor and acts by binding to the active site of FXa preventing substrate binding. Factor Xa is responsible for the conversion of prothrombin to thrombin in the coagulation cascade [25]. Rivaroxaban has a rapid onset of action, reaching maximum plasma concentrations after 2-3 hours and has a half-life of 8–10 hours [25, 26]. No increase in maximum plasma concentrations has been demonstrated in the obese patients (>120 kg). However, the maximum plasma concentration has been demonstrated to be up to 24% higher in patients weighing less than 50 kg [27]. Rivaroxaban is metabolized in the liver and primarily excreted renally (66%), with the remainder excreted through the gastrointestinal tract [25]. In patients with impaired renal function, the clearance of rivaroxaban is decreased moderately, and its use is not recommended for patients with severe renal impairment (creatinine clearance < 30 mL/min) [26, 28]. 

## 3. Phase III Clinical Trials of Rivaroxaban versus Enoxaparin

The safety and efficacy of rivaroxaban has been assessed in two phase III trials each for patients undergoing THA and TKA [18–21] (Table 1). All four were randomized, double-blind, placebo-controlled studies. The study group responsible for these trials is the Regulation of Coagulation in Orthopedic Surgery to Prevent Deep Venous Thrombosis and Pulmonary Embolism (RECORD). The primary efficacy outcome for each study was DVT (symptomatic or asymptomatic venographic), PE, or death, and the primary safety outcome was major bleeding. Major bleeding was defined as bleeding that was fatal, occurred in a critical organ, requiring reoperation, or was clinically overt with a drop in the hemoglobin level of at least 2 g/dL or requiring at least 2 units of blood transfusion. Major bleeding did not include surgical site bleeding unless it led to reoperation. Clinically relevant nonmajor bleeding was a secondary safety outcome and was defined as multiple-source bleeding, spontaneous hematoma greater than 25 cm^2^, excessive wound hematoma, nose bleeding >5 minutes, gingival bleeding >5 minutes, macroscopic hematuria, rectal bleeding, coughing or vomiting blood, vaginal bleeding, blood in semen, intra-articular bleeding with trauma, or surgical site bleeding.

The RECORD 1 trial included 4,541 patients undergoing THA that were assigned to received either rivaroxaban 10 mg daily or enoxaparin 40 mg daily, plus either a placebo tablet or injection. Rivaroxaban was started after surgery, and enoxaparin was started the evening prior to surgery. The duration of treatment was 33 days on average in both groups. The primary efficacy outcome occurred in 1.1% of patients in the rivaroxaban group and in 3.7% of patients in the enoxaparin group (*P* < 0.001). Major bleeding occurred in 0.3% of patients in the rivaroxaban group and in 0.1% of patients in the enoxaparin group (*P* = 0.018). The combined rate of major and clinically relevant non-major bleeding was 3.2% in the rivaroxaban group and 2.5% in the enoxaparin group, which was not statistically significant [18].

The RECORD 2 trial included 2,509 patients undergoing THA that were assigned to receive either rivaroxaban 10 mg daily for 31–39 days or enoxaparin 40 mg daily for 10–14 days. The primary efficacy outcome occurred in 2.0% of patients in the rivaroxaban group and in 9.3% of patients in the enoxaparin group (*P* < 0.0001). Major bleeding occurred in only 1 patient in each group. However, the rate of clinically relevant non-major bleeding occurred in 6.5% of patients in the rivaroxaban group compared to 5.5% of patients in the enoxaparin group [19].

The RECORD 3 trial included 2,531 patients undergoing TKA that were assigned to receive either rivaroxaban 10 mg daily for or enoxaparin 40 mg daily for 10–14 days. The primary efficacy outcome occurred in 9.6% of patients in the rivaroxaban group and in 18.9% of patients in the enoxaparin group (*P* < 0.001). Major bleeding occurred in 0.6% of patients in the rivaroxaban group and 0.5% of patients in the enoxaparin group. The combined incidence of major and clinically relevant non-major bleeding was 3.3% in the rivaroxaban group and 2.7% in the enoxaparin group, which was not statistically significant [20].

The RECORD 4 trial included 3,148 patients undergoing TKA that were assigned to receive either rivaroxaban 10 mg daily for or enoxaparin 30 mg twice daily for 10–14 days. The primary efficacy outcome occurred in 6.9% of patients in the rivaroxaban group and in 10.1% of patients in the enoxaparin group (*P* = 0.0118). Major bleeding occurred in 0.7% of patients in the rivaroxaban group and 0.3% of patients in the enoxaparin group (*P* = 0.1096). The combined incidence of major and clinically relevant non-major bleeding was 3.0% in the rivaroxaban group and 2.3% in the enoxaparin group [21].

## 4. Pooled Data from Clinical Trials

Pooled analyses of the data from the RECORD trials have demonstrated similar findings of increased efficacy of rivaroxaban at all time intervals [29, 30]. Moreover, patients in the rivaroxaban groups had a reduced rate of the composite of death, myocardial infarction, and stroke in addition to symptomatic VTE [29]. With data pooled from all 4 RECORD trials, rivaroxaban significantly reduced the rate of symptomatic VTE and death over the total study duration (0.81% versus 1.63%, *P* < 0.001; HR 0.42, 95%CI 0.29–0.63) [29, 30]. Due to the discrepancy in treatment duration of the RECORD 2 trial (enoxaparin given for 10–14 days versus rivaroxaban given for 31–39 days), other authors have excluded this study when pooling data. Despite this, rivaroxaban still demonstrated superior efficacy compared to enoxaparin for the primary outcome (RR 0.5, 95%CI 0.34–0.73, *P* = 0.0003) [31]. 

**Figure 1:**
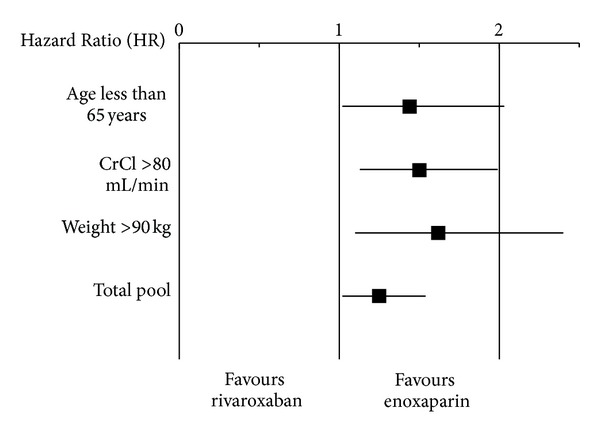
Pooled incidence of major plus clinically relevant non-major bleeding for RECORD trials 1–4. Patients taking rivaroxaban had more events than patients taking enoxaparin (*P* = 0.03). In subgroup analysis, more events occurred in patients taking rivaroxaban less than 65 years of age (*P* = 0.04), weighing less than 90 kg (*P* = 0.02), and with creatinine clearance over 80 mL/min (*P* = 0.005) [30].

However, in each of the RECORD trials, the rate of bleeding complications was higher in the rivaroxaban group, although not statistically significant. Pooled data from all 4 trials demonstrated a trend of higher rates of major bleeding in the rivaroxaban group for the treatment period (0.39% versus 0.21%, *P* = 0.076). The combined endpoint of major plus clinically relevant non-major bleeding was significantly higher in the rivaroxaban group during the treatment period (3.19% versus 2.55%, *P* = 0.039; HR 1.25, 95%CI 1.01–1.54). In subgroup analysis, significantly higher rates of major plus clinically relevant nonmajor bleeding were also seen in patients under 65 years old (HR 1.44, 95%CI 1.02–2.04), patients weighing greater than 90 kg (HR 1.62, 95%CI 1.09–2.4) and patients with creatinine clearance >80 mL/min (HR 1.5, 95%CI 1.12–2.00) [23, 29, 30] (Figure 1). Similarly, with the RECORD 2 trial excluded, pooled analysis demonstrated higher rates of major plus clinically relevant non-major bleeding in patients taking rivaroxaban (RR 1.29, 95%CI 1.03–1.63) [31]. Another study pooling data from the RECORD trials that excluded RECORD 2 demonstrated a lower risk of major plus non-major clinically relevant bleeding in the enoxaparin group (OR 0.79, 95%CI 0.62–0.99, *P* = 0.049) [22]. The number needed to harm by treating with rivaroxaban in this study was 167 patients [22].

In addition to bleeding, rivaroxaban was also associated with increased risk for other adverse events. Pooled data demonstrated that patients on rivaroxaban had a higher rate of serious on-treatment adverse events (OR 1.27, 95%CI 1.09–1.48, *P* = 0.002) and also had a higher rate of treatment discontinuation due to adverse events (OR 1.22, 95%CI 1.00–1.49, *P* = 0.05). Furthermore, patients in the rivaroxaban group were more likely to have alanine aminotransferase (ALT) levels more than 3x the upper limit of normal during treatment (OR1.42, 95%CI 1.06–1.88, *P* = 0.02) [22].

## 5. Conclusion

Venous thromboembolism is a common complication after THA and TKA and creates a substantial burden to patients, providers, and costs to the healthcare system. Currently available anticoagulants have limitations that lead to decreased compliance with VTE prophylaxis guidelines. Rivaroxaban has superior efficacy compared to enoxaparin for the prevention of symptomatic VTE for patients undergoing THA and TKA. Although the patient compliance rate while taking rivaroxaban remains unknown, rivaroxaban is administered orally with fixed dosing and does not require monitoring which makes it attractive. Furthermore, there is evidence to support that the patients receiving rivaroxaban experience a reduced rate of the composite of death, myocardial infarction, and stroke in addition to the reduction in symptomatic VTE. However, the bleeding risk with rivaroxaban after THA and TKA is not fully understood. Pooled data from the phase III clinical trials indicate that patients taking rivaroxaban experience more bleeding complications than those patients taking Lovenox. Moreover, a retrospective cohort study of patients undergoing THA and TKA found that those patients that received thromboprophylaxis with rivaroxaban were more likely to return to the operating room than those patients that received thromboprophylaxis with LMWH [32]. Another large retrospective cohort study comparing rivaroxaban to LMWH after THA and TKA found that patients taking rivaroxaban were less likely to experience bleeding, return to the operating room for wound complications, and had a shorter hospital stay [33]. Specific patient groups at increased risk of bleeding complications include those patients under 65 years old, patients weighing greater than 90 kg, and patients with creatinine clearance >80 mL/min. Postmarketing surveillance is critical to continue to monitor and establish the safety profile of this agent in larger and more diverse patient populations.

## Figures and Tables

**Table 1:** Description of RECORD trials comparing rivaroxaban to enoxaparin for VTE prophylaxis in patients undergoing THA and TKA.

Procedure	RECORD 1	RECORD 2	RECORD 3	RECORD 4
	THA	THA	TKA	TKA
Rivaroxaban dose	10 mg daily	10 mg daily	10 mg daily	10 mg daily
Enoxaparin dose	40 mg daily	40 mg daily	40 mg daily	30 mg BID
Treatment duration (days)	31–39	Enoxaparin 10–14, rivaroxaban 31–39	10–14	10–14
Patients (*n*)	4541	2509	2531	3148
